# Dynamic white blood cell kinetics enable early risk stratification of differentiation syndrome in low- and intermediate-risk acute promyelocytic leukemia

**DOI:** 10.3389/fonc.2026.1961271

**Published:** 2026-09-18

**Authors:** Jiajuan Ji, Yuting Zhu, Dan Luo, Peiwen Ding, Huibo Li, Yanqiu Zhao, Zhibo Guo, Qi Li, Jie Liu, Dan Guo, Yujuan Gao, Shengjin Fan

**Affiliations:** 1 Department of Hematology, The First Affiliated Hospital of Harbin Medical University, Harbin, China; 2 National Health Commission (NHC) Key Laboratory of Cell Transplantation, the First Affiliated Hospital, Harbin Medical University, Harbin, China; 3 Key Laboratory of Blood and Hematopoietic System in Heilongjiang, Harbin, China

**Keywords:** acute promyelocytic leukemia, differentiation syndrome, leukocyte dynamics, nomogram, proliferation rate

## Abstract

**Background:**

Differentiation syndrome (DS) is a potentially life-threatening complication during induction therapy for acute promyelocytic leukemia (APL). Previous studies have linked white blood cell (WBC) elevation with DS development; however, conventional WBC-based assessment mainly relies on peak values and does not adequately reflect the dynamic pattern or timing of WBC changes. We aimed to develop and internally validate an early warning model for DS based on early WBC kinetics during arsenic trioxide (ATO) induction therapy in patients with low- and intermediate-risk APL.

**Methods:**

We retrospectively analyzed 224 newly diagnosed patients with low- and intermediate-risk APL who received single-agent ATO induction therapy. Early WBC kinetics and baseline clinical parameters were analyzed using least absolute shrinkage and selection operator regression and multivariate logistic regression to construct a nomogram.

**Results:**

DS occurred in 90 patients (40.2%), with a median onset time of 8 days (interquartile range, 7–11 days). WBC doubling occurred at a median of 3 days before DS onset, whereas the WBC peak occurred at a median of 2.5 days after DS onset. Rapid WBC proliferation and increased peripheral blood promyelocyte percentage were independent risk factors, whereas prolonged WBC doubling time was associated with reduced DS risk. The nomogram showed good discrimination, with an area under the curve of 0.825 (95% confidence interval, 0.770–0.880); the corrected area under the curve remained 0.817 after bootstrap internal validation.

**Conclusion:**

Early WBC kinetic characteristics provide time-sensitive indicators for DS risk assessment. This model may assist with early DS risk stratification and monitoring in low- and intermediate-risk APL patients.

## 1 Introduction

Acute promyelocytic leukemia (APL) was historically considered one of the most fatal subtypes of acute leukemia. However, the development of differentiation therapy with all-trans retinoic acid (ATRA) and arsenic trioxide (ATO) has dramatically changed the treatment landscape for APL. Current therapeutic strategies achieve complete remission rates exceeding 90% (1), with 5-year overall survival rates above 80% (2, 3). Despite these advances, treatment failure due to early death still occurs in approximately 5–15% of patients (3–5). According to peer-reviewed data, hemorrhage (57%) is the primary cause of early death, followed by sepsis (18%), differentiation syndrome (DS, 12%), and thrombotic events (2%) (5, 6). The reported incidence of DS varies considerably, ranging from 2% to 50% (1, 7–10), which may be attributed to differences in diagnostic criteria, preventive strategies, and treatment approaches among studies (1, 9).

DS is a characteristic inflammatory complication occurring during APL induction therapy. Its development involves the infiltration of abnormal differentiating cells, inflammatory cytokine release, and capillary leakage (11, 12). In the absence of specific laboratory biomarkers, DS diagnosis mainly relies on clinical manifestations, including dyspnea, unexplained fever, weight gain exceeding 5 kg, unexplained hypotension, acute renal failure, and radiological evidence of pulmonary infiltration, pleural effusion, or pericardial effusion (13). Its clinical presentation overlaps substantially with that of sepsis, capillary leak syndrome, pneumonia, and other complications, frequently leading to missed or delayed diagnosis and overdiagnosis. Therefore, the early identification of patients at high risk of DS before the development of overt clinical manifestations is important for timely intervention and optimal patient management.

To improve the early recognition of DS, previous studies have reported several clinical and laboratory factors associated with DS development. Post-treatment white blood cell (WBC) peak count, body mass index (BMI), serum creatinine (Cr) levels, and treatment regimens have been associated with DS risk (7, 14–19). Among these factors, dynamic WBC elevation during treatment (14, 20), particularly peak WBC values, has been considered closely associated with DS development. However, conventional WBC peak–based assessment relies on relatively late-occurring indicators, which may appear after the onset of clinical manifestations of DS and therefore limit its value for early risk assessment. Our previous study demonstrated that dynamic changes in WBC doubling may precede the onset of clinical DS and provide potential early warning information (21).

Although several DS-related risk factors have been identified, individualized early risk assessment models based on dynamic indicators remain limited. In clinical practice, patients with high-risk APL and initial hyperleukocytosis usually receive cytoreductive therapy and prophylactic corticosteroids to reduce the risk of DS. However, for patients with low- and intermediate-risk APL, whether prophylactic corticosteroids are required and when cytoreductive therapy should be initiated according to dynamic disease changes remain unclear (22). Therefore, we aimed to characterize early WBC kinetic patterns during ATO induction therapy and develop and internally validate an early warning model for DS risk stratification in patients with low- and intermediate-risk APL.

## 2 Methods

### 2.1 Study design and patient selection

A total of 323 patients with newly diagnosed APL at The First Affiliated Hospital of Harbin Medical University between January 2018 and January 2026 were included in this single-center retrospective cohort study. All cases were genetically confirmed by the detection of the t (15, 17) translocation or PML-RARα fusion gene. Patients were stratified into three risk groups according to the Sanz criteria (23): low-risk (baseline WBC ≤ 10×10^9^/L and platelet [PLT] ≥40×10^9^/L), intermediate-risk (WBC ≤ 10×10^9^/L and PLT < 40×10^9^/L), and high-risk (WBC > 10×10^9^/L). The study protocol was approved by the Institutional Ethics Committee of Harbin Medical Hospital. Given the retrospective design, the requirement for informed consent to participate in the study was waived.

Patients were considered eligible if they were newly diagnosed with low- or intermediate-risk APL and were treated with single-agent ATO induction therapy. Patients were excluded for prior antileukemic treatment, > 25% missing key clinical data, concomitant malignancies, severe hepatic or renal dysfunction, pregnancy or lactation, and death within the first 14 days after ATO initiation that precluded completion of early WBC kinetic and DS assessment. After rigorous screening, 224 patients were included in the final cohort, with 90 DS events, providing an adequate number of outcome events for model development.

### 2.2 Treatment protocol

All patients with suspected APL were treated immediately immediate single-agent ATO induction therapy at a daily dose of 0.16 mg/kg, with a maximum daily dose of 10 mg. Cytotoxic chemotherapy was administered at the clinicians’ discretion according to baseline and dynamic WBC changes to reduce tumor burden and mitigate the risk of DS. Standardized blood product support was provided to all patients to maintain PLT counts ≥ 30×10^9^/L, hemoglobin (HGB) ≥ 70 g/L, and plasma fibrinogen (FIB) ≥ 1.5 g/L (24). All treatments were delivered in accordance with routine clinical practice. Written informed consent for ATO-based treatment was obtained from all patients before therapy initiation.

### 2.3 Definitions and grouping

DS was diagnosed according to Frankel et al. (13, 25) based on core clinical manifestations, including dyspnea, unexplained fever, weight gain exceeding 5 kg, unexplained hypotension, acute renal failure, pulmonary infiltration, pleural effusion, and pericardial effusion. DS severity was stratified by the number of presenting symptoms: severe DS was defined as the presence of ≥ 4 symptoms, moderate DS as 2–3 symptoms, and isolated single symptoms were classified as indeterminate and were not included in the DS group. Early mortality was defined as all-cause death within 30 days after ATO initiation.

To characterize early leukocyte kinetic changes during ATO-based induction therapy, serial WBC counts were collected daily during the first 14 days after ATO initiation. Individual WBC trajectories were evaluated to identify patterns of rapid leukocyte rebound after reaching the nadir WBC value.

For each patient, the current nadir WBC value was dynamically determined at each assessment time point and defined as the lowest WBC count observed from the initiation of ATO therapy up to that day. The increase in WBC from the current nadir was subsequently assessed using absolute and time-adjusted relative kinetic parameters.

Based on the exploratory analysis of individual WBC trajectories and receiver operating characteristic (ROC) curve analysis with the Youden index, a rapid WBC proliferation indicator was established. Considering the different characteristics of absolute leukocyte burden and relative growth kinetics, two complementary criteria were applied:

1. Absolute increase pattern: When the daily WBC count was ≥ 15 × 10^9^/L, a rapid proliferation event was defined as an increase of > 10 × 10^9^/L compared with the current nadir WBC value.
2. Relative increase pattern: When the daily WBC count was < 15 × 10^9^/L, the relative WBC growth velocity was calculated as: Relative WBC growth velocity = (WBC_day_-WBC_nadir_)/WBC_nadir_/Δt.

where WBC_day_ was defined as the WBC count on the assessment day, WBC_nadir_ was defined as the current nadir WBC value, and Δt was defined as the number of days between the assessment day and the day on which the nadir WBC occurred. A relative growth velocity of ≥ 2 was considered indicative of rapid proliferation. To assess the individual associations of the two definitions with DS, the absolute-increase and relative-growth definitions were also evaluated separately across the entire cohort, irrespective of the WBC cutoff.

Patients were classified as having rapid proliferation if at least one daily assessment during the 14-day observation period fulfilled either criterion. A cutoff value of 15 × 10^9^/L was used as a kinetic classification threshold to distinguish absolute leukocyte accumulation from relative growth velocity patterns rather than as a diagnostic threshold for DS.

### 2.4 Clinical data collection

Clinical data were retrospectively extracted from electronic medical records, including patients’ demographic characteristics, baseline laboratory parameters, bone marrow (BM) and peripheral blood (PB) promyelocyte percentages, cytogenetic features, and serum biochemical indices. Dynamic WBC profiles within the initial 14 days of induction therapy, WBC doubling parameters, WBC peak values, and biochemical data at DS onset were also collected. All data were independently recorded and cross-checked by two researchers, with inconsistencies resolved by reference to the original medical records. Missing baseline variables and serial WBC measurements from days 2–14 after ATO initiation were handled using multiple imputation by chained equations. Five imputed datasets were generated, with 20 iterations performed for each imputation chain. Imputation methods were specified according to variable type: continuous variables were imputed using predictive mean matching, whereas binary categorical variables were imputed using logistic regression. After imputation, dynamic WBC parameters were derived from the reconstructed WBC trajectories. The distribution of missing values before multiple imputation is presented in **Table 1**. Missing data were mainly observed in serial WBC measurements during early ATO induction and in baseline laboratory variables, with missingness generally below 10%.

**Table 1:** Missing data distribution of variables before multiple imputation.

Variable	Missing (n)	Missing (%)
WBC day 11 after ATO initiation	23	10.27
WBC day 13 after ATO initiation	22	9.82
WBC day 10 after ATO initiation	21	9.38
WBC day 9 after ATO initiation	19	8.48
Height	18	8.04
WBC day 12 after ATO initiation	18	8.04
ALT at WBC doubling	17	7.59
AST at WBC doubling	17	7.59
WBC day 2 after ATO initiation	16	7.14
WBC day 7 after ATO initiation	16	7.14
WBC day 8 after ATO initiation	15	6.70
WBC day 6 after ATO initiation	14	6.25
WBC day 14 after ATO initiation	14	6.25
Neut	13	5.80
Mono	13	5.80
WBC day 3 after ATO initiation	13	5.80
Baseline lymphocyte count	12	5.36
PB	11	4.91
WBC day 5 after ATO initiation	11	4.91
WBC day 4 after ATO initiation	10	4.46
Cr	8	3.57
WBC doubling time	6	2.68
BM	5	2.23
Body weight	2	0.89
APTT	2	0.89
PT	1	0.45

ALT, alanine aminotransferase; APTT, activated partial thromboplastin time; AST, aspartate aminotransferase; BM, bone marrow promyelocyte percentage; Cr, creatinine, Mono, monocyte, Neut ,neutrophil; PB, peripheral blood promyelocyte percentage; PT, prothrombin time; WBC, white blood cell. Missing values were assessed before multiple imputation. Baseline variables and serial WBC measurements from day 2 to day 14 after initiation of ATO therapy were handled using multiple imputation by chained equations (MICE). Dynamic WBC-related parameters were subsequently derived from the imputed datasets.

### 2.5 Statistical analysis

All statistical analyses were performed using R version 4.6.0. Continuous variables conforming to a normal distribution were presented as mean ± standard deviation [ $\bar{x}$ ± sd] and compared using the independent samples t-test, whereas non-normally distributed data were expressed as median (interquartile range) [M (P25, P75)] and compared using the Mann-Whitney U test. Categorical variables were summarized as frequencies and percentages, with group differences assessed by the χ^2^ test. ROC curves were constructed to evaluate the predictive performance of WBC-related indices for DS occurrence; optimal cutoff values were determined using the maximum Youden index. To reduce variable redundancy, mitigate the impact of multicollinearity, and minimize the risk of model overfitting, univariate binary logistic regression analyses were initially performed to explore potential associations between candidate variables and DS occurrence. Variables with potential predictive value were subsequently entered into the least absolute shrinkage and selection operator (LASSO) regression model for dimensionality reduction and variable selection. The penalty coefficient was determined according to the one-standard-error criterion (lambda.1se) derived from cross-validation, allowing the selection of stable core predictors while avoiding excessive model complexity. Variables identified by LASSO regression were further incorporated into a multivariable stepwise logistic regression model to establish an early warning model for DS. Multicollinearity among variables included in the final multivariable logistic regression model was assessed using variance inflation factors (VIFs). Internal validation was performed using bootstrap resampling with 1,000 repetitions to evaluate model stability and estimate optimism-corrected performance. Model discrimination was assessed using the area under the receiver operating characteristic curve (AUC) with 95% confidence intervals (CIs). Calibration performance was evaluated using bootstrap-calibrated calibration curves; decision curve analysis (DCA) was performed to assess the clinical net benefit of the model. A two-tailed P value < 0.05 was considered significant.

## 3 Results

### 3.1 Baseline demographic, clinical, and laboratory characteristics

A total of 224 patients (104 males and 120 females) were included in this retrospective analysis. DS developed in 40.2% of the cohort. Baseline demographic, clinical, and laboratory characteristics are summarized in **Table 2**. No statistically significant differences were observed between the DS and non-DS groups with respect to sex, age, BMI, or body surface area (BSA) (all P > 0.05). At diagnosis, no significant intergroup differences were detected in HGB, PLT, or absolute lymphocyte count. In contrast, compared with the non-DS group, the DS group exhibited significantly higher absolute WBC counts (1.76 × 10^9^/L vs. 1.16 × 10^9^/L), absolute neutrophil counts (0.44 × 10^9^/L vs. 0.28 × 10^9^/L), and absolute monocyte counts (0.28 × 10^9^/L vs. 0.11 × 10^9^/L) (all P < 0.005). Coagulation dysfunction was more pronounced in the DS group than in the non-DS group, as evidenced by significantly lower FIB levels (1.35 g/L vs. 1.70 g/L), prolonged prothrombin time (PT) (13.6 seconds vs. 13.1 seconds), elevated D-dimer (DD) concentrations (21.41 mg/L FEU vs. 12.91 mg/L FEU), higher disseminated intravascular coagulation (DIC) scores (5.04 vs. 4.57), and a greater proportion of patients meeting the diagnostic criteria for overt DIC (85.7% vs. 65.4%) (all P < 0.05). No significant differences were observed between the groups in hepatic or renal function parameters or the incidence of bleeding complications at diagnosis. However, markers of tumor burden, including promyelocyte percentages in BM and PB, were significantly elevated in the DS group (all P < 0.001).

**Table 2:** Comparison of baseline characteristics in patients with or without DS.

Characteristics	All patients (n=224)	APLs without DS (n=134)	APLs with DS (n=90)	χ^2^/Z/t value	P value
Gender (n/%,Male/Female)	104(46.4%)/120(53.6%)	61(45.5%)/73(54.5%)	43(47.8%)/47(52.2%)	0.038	0.8452
Age [year,M(P_25_,P_75_)]	47<(>34-58<)>	46(35-57.75)	48.5(34-58)	-0.421	0.6747
BMI [M(P_25_,P_75_)]	23.55(21.48-26.36)	23.77(21.43-26.69)	23.19(21.8-26.20)	0.793	0.4285
BSA [M(P_25_,P_75_)]	1.74(1.62-1.87)	1.74(1.62-1.87)	1.74(1.60-1.86)	0.442	0.6595
BM [%,M(P_25_,P_75_)]	77.50(66.00-86.50)	75.25(62.62-84.45)	81.00(71.75-88.00)	-3.443	**0.0006**
PB [%,M(P_25_,P_75_)]	15.50(2.00-42.25)	9.50(1.25-36.00)	35.00(10.25-58.50)	-4.72	**<0.0001**
WBC [×10^9^/L,M(P_25_,P_75_)]	1.30(0.85-3.24)	1.16(0.74-2.08)	1.76(1.14-4.55)	-4.466	**<0.0001**
Neut [×10^9^/L,M(P_25_,P_75_)]	0.34(0.17-0.74)	0.28(0.14-0.68)	0.44 (0.23-1.12)	-2.9	**0.0038**
Lymph [×10^9^/L,M(P_25_,P_75_)]	0.58(0.36-0.85)	0.56(0.36-0.80)	0.61(0.39-0.97)	-1.81	0.0710
Mono [×10^9^/L,M(P_25_,P_75_)]	0.15(0.05-0.51)	0.11(0.04-0.33)	0.28(0.07-0.84)	-3.17	**0.0016**
HGB [g/L,M(P_25_,P_75_)]	76.00(62.00-96.00)	75.62(62.37-94.75)	77.66(62.00-99.64)	-0.413	0.6802
PLT [×10^9^/L,M(P_25_,P_75_)]	28.99(16.00-48.44)	29.2(17.32-54.52)	25.7(15.33-43.67)	1.263	0.2070
FIB [g/L,M(P_25_,P_75_)]	1.59(1.07-2.51)	1.70(1.11-2.61)	1.35(0.88-2.34)	-2.37	**0.0176**
PT [s,M(P_25_,P_75_)]	13.20(12.47-14.40)	13.1(12.33-14.1)	13.60(12.62-14.88)	-2.97	**0.0030**
APTT [s,M(P_25_,P_75_)]	26.30(24.48-28.85)	26.05(24.40-27.90)	26.75(24.72-29.70)	-1.24	0.2139
DD [mg/L,M(P_25_,P_75_)]	14.36(7.37-29.96)	12.91(6.28-22.97)	21.41(8.41-34.04)	-2.87	**0.0040**
ALT [U/L,M(P_25_,P_75_)]	20.65(12.90-31.35)	20.20(11.93-29.52)	21.35(14.15-33.75)	-1.465	0.1433
AST [U/L,M(P_25_,P_75_)]	20.05(14.15,27.85)	19.65(13.85,26.23)	21.40(14.70,30.00)	-1.328	0.1845
Cr [μmol/L,M(P_25_,P_75_)]	64.20(54.70-76.45)	62.40(53.55-75.40)	65.95(56.65-79.20)	-1.322	0.1866
DIC score [ $\bar{x}$ ± sd]	4.76 ± 1.09	4.57 ± 1.07	5.04 ± 1.05	3.310	**0.0011**
DIC (n/%,Yes/No)	157(70.10)/67(29.9)	85(63.40)/49(36.60)	72(85.70)/18(14.30)	6.281	**0.0122**
Bleeding (n/%,Yes/No)	36(16.10%)/188(83.90%)	17(12.70%)/117(87.30%)	19(21.10%)/71(78.90%)	2.243	0.1342

ALT, alanine aminotransferase; APTT, activated partial thromboplastin time; AST,aspartate aminotransferase; BM, bone marrow promyelocyte percentage; BMI, body mass index; BSA, body surface area; Cr, creatinine; DD, D-dimer concentrations; DIC, disseminated intravascular coagulation; DS, differentiation syndrome; FIB, fibrinogen; HGB, hemoglobin; Lymph,lymphocyte; Mono, monocyte; Neut,neutrophil; PB, peripheral blood promyelocyte percentage; PLT, platelet; PT, prothrombin time; WBC, white blood cell.

Bold values indicate statistical significance (P < 0.05).

### 3.2 Clinical characteristics and temporal distribution of DS

Among the 224 patients included in this study, 90 patients developed DS during induction therapy with ATO. According to the predefined symptom-based severity criteria, 72 patients (80.0%) were classified as moderate DS and 18 patients (20.0%) as severe DS. The clinical manifestations of DS are summarized in **Figure 1**. Unexplained fever was the most frequent manifestation, occurring in 85 patients (94.4%), followed by dyspnea in 71 patients (78.9%). Other common manifestations included weight gain ≥ 5 kg (37 patients, 41.1%) and pericardial/pleural effusion (35 patients, 38.9%). Less frequent manifestations included hypotension (11 patients, 12.2%), lung infiltration (six patients, 6.7%), and acute renal failure (one patient, 1.1%). The temporal distribution of DS onset after ATO is shown in **Figure 1**. DS predominantly occurred during the early induction phase, with a median onset time of 8 days after treatment initiation (interquartile range, 7–11 days). These findings highlight the importance of the early identification of patients at high risk for DS during the initial phase of ATO therapy. Early mortality during initial treatment was further evaluated according to DS status. Among the 224 patients, early death occurred in 6 of 90 patients (6.7%) with DS and in 3 of 134 patients (2.2%) without DS. Although the incidence of early mortality was numerically higher in patients with DS than in those without DS, the difference did not reach statistical significance (Fisher’s exact test, P = 0.162). In logistic regression analysis, DS occurrence showed a trend toward an increased risk of early mortality (odds ratio [OR], 3.12; 95% CI, 0.80–15.09), although the association was not statistically significant.

**Figure 1:**
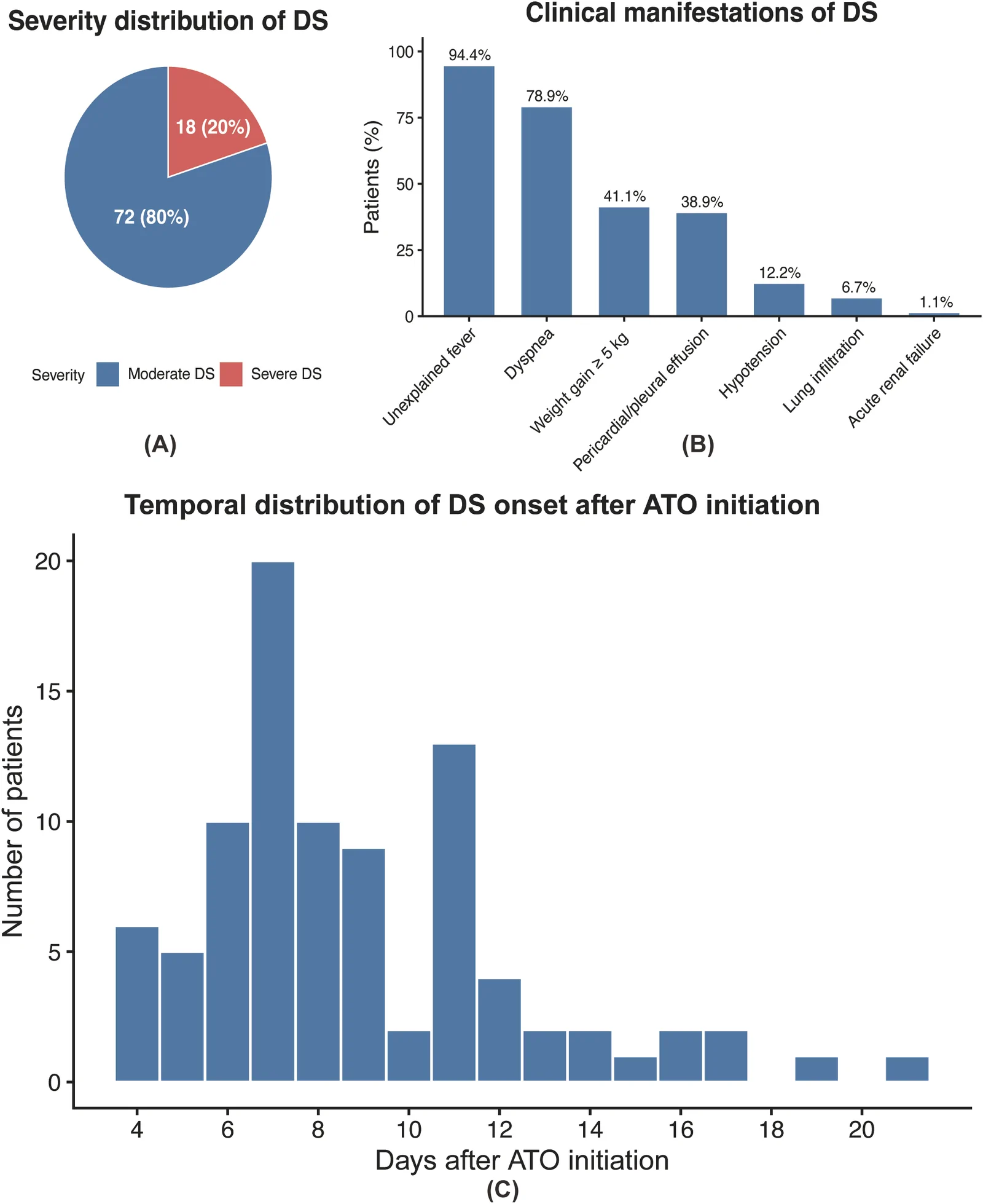
Clinical characteristics and temporal patterns of differentiation syndrome after arsenic trioxide induction in APL patients **(A)** Distribution of DS severity according to the predefined symptom-based criteria. Moderate DS was defined as the presence of 2–3 DS-related clinical manifestations, whereas severe DS was defined as ≥4 manifestations. Among the 90 patients with DS, 72 (80.0%) were classified as moderate DS and 18 (20.0%) as severe DS. **(B)** Frequency of individual clinical manifestations among patients with DS. Unexplained fever and dyspnea were the most common manifestations, followed by weight gain ≥5 kg and pericardial/pleural effusion. Other less frequent manifestations included hypotension, lung infiltration, and acute renal failure. **(C)** Temporal distribution of DS onset after arsenic trioxide initiation. The median time from ATO initiation to DS onset was 8 days (interquartile range, 7–11 days), indicating that DS predominantly occurred during the early induction phase.

### 3.3 Univariate analysis of risk factors for DS

The absolute-increase and relative-growth definitions were separately evaluated across the entire cohort. Both definitions were significantly associated with DS occurrence, with ORs of 10.92 (95% CI, 5.42–22.02; P < 0.001) and 2.33 (95% CI, 1.34–4.07; P = 0.003), respectively. Based on the observed WBC trajectory patterns, an exploratory cutoff of 15 × 10^9^/L was used to combine the two definitions to capture rapid WBC changes across different WBC levels. The resulting rapid WBC proliferation indicator was significantly associated with an increased risk of DS (OR, 7.33; 95% CI, 3.87–14.73; P < 0.0001). Conversely, an extended WBC doubling time (OR, 0.74 per day; 95% CI, 0.66–0.82; P < 0.0001) and a delayed WBC peak (OR, 0.90 per day; 95% CI, 0.86–0.94; P < 0.0001) were associated with reduced DS risk. Using the Youden index–optimized cutoff method, the optimal dichotomization thresholds were determined to be 6.5 days for WBC doubling time and 7.54 × 10^9^/L for WBC count, yielding Youden indices of 0.416 and 0.354, respectively. Stratification by WBC doubling time (> 6.5 days vs. ≤ 6.5 days) yielded two groups, rapid versus non-rapid doubling, which were significantly associated with DS (OR 6.08, 95% CI 3.37–11.33; P < 0.0001). Similarly, stratification by WBC count (> 7.54 10^9^/L vs. ≤ 7.54 × 10^9^/L) identified high- versus low-WBC-load subgroups, which were also significantly associated with DS (OR 5.04, 95% CI 2.88–10.64; P < 0.0001). Furthermore, pretreatment coagulopathy (specifically overt DIC at initial diagnosis) was associated with more than double the odds of DS (OR 2.31, 95% CI 1.25–4.39; P = 0.008). Each additional second of PT conferred a 36% increase in DS risk (P = 0.001); each one-point increment in the DIC score was associated with a 57% increase in DS risk (P = 0.002). Additional significant predictors included baseline WBC count, absolute monocyte count, WBC count at doubling time, WBC peak count, BM promyelocyte percentage, and PB promyelocyte percentage. The full results are presented in **Table 3**.

**Table 3:** Results of univariate logistic regression analysis.

Variable	OR	95% CI	P value
Rapid proliferation	7.33	3.87-14.73	**<0.0001**
WBC double group	6.08	3.37-11.33	**<0.0001**
WBC double days	0.74	0.66-0.82	**<0.0001**
WBC _peak_	1.06	1.04-1.09	**<0.0001**
WBC high cut	5.40	2.88-10.64	**<0.0001**
PB	1.02	1.01-1.04	**<0.0001**
WBC _double_	1.15	1.08-1.23	**<0.0001**
WBC peak days	0.90	0.86-0.94	**<0.0001**
WBC _baseline_	1.32	1.16-1.52	**<0.0001**
DIC score	1.57	1.20-2.11	**0.0018**
PT _baseline_	1.36	1.13-1.66	**0.0018**
BM	1.02	1.01-1.05	**0.0038**
Mono _baseline_	1.64	1.20-2.37	**0.0044**
DIC	2.31	1.25-4.39	**0.0088**
ALT _WBC double_	0.99	0.99-1.00	0.0561
DD _baseline_	1.01	1.00-1.02	0.0691
Cr _WBC double_	1.01	1.00-1.03	0.0915
FIB _baseline_	0.89	0.72-1.08	0.2681
AST _WBC double_	1.00	0.99-1.00	0.3291
Neut _baseline_	0.99	0.90-1.07	0.8684

ALT, alanine aminotransferase; AST, aspartate aminotransferase; BM, bone marrow promyelocyte percentage; Cr, creatinine; DD, D-dimer concentrations; DIC, disseminated intravascular coagulation; FIB, fibrinogen; Mono, monocyte; Neut, neutrophil; PB, peripheral blood promyelocyte percentage; PLT, platelet; PT, prothrombin time; WBC, white blood cell. Bold values indicate statistical significance (P < 0.05).

### 3.4 Timing relationship between WBC kinetics and DS onset

We further analyzed the time intervals from WBC doubling and WBC peak to DS onset in patients with DS. The results showed that WBC doubling consistently preceded DS onset, with a median advance of 3 days, whereas the WBC peak levels occurred subsequent to DS onset, with a median delay of 2.5 days (**Figure 2**). Therefore, the WBC peak was not considered a candidate early predictive variable in subsequent modeling.

**Figure 2:**
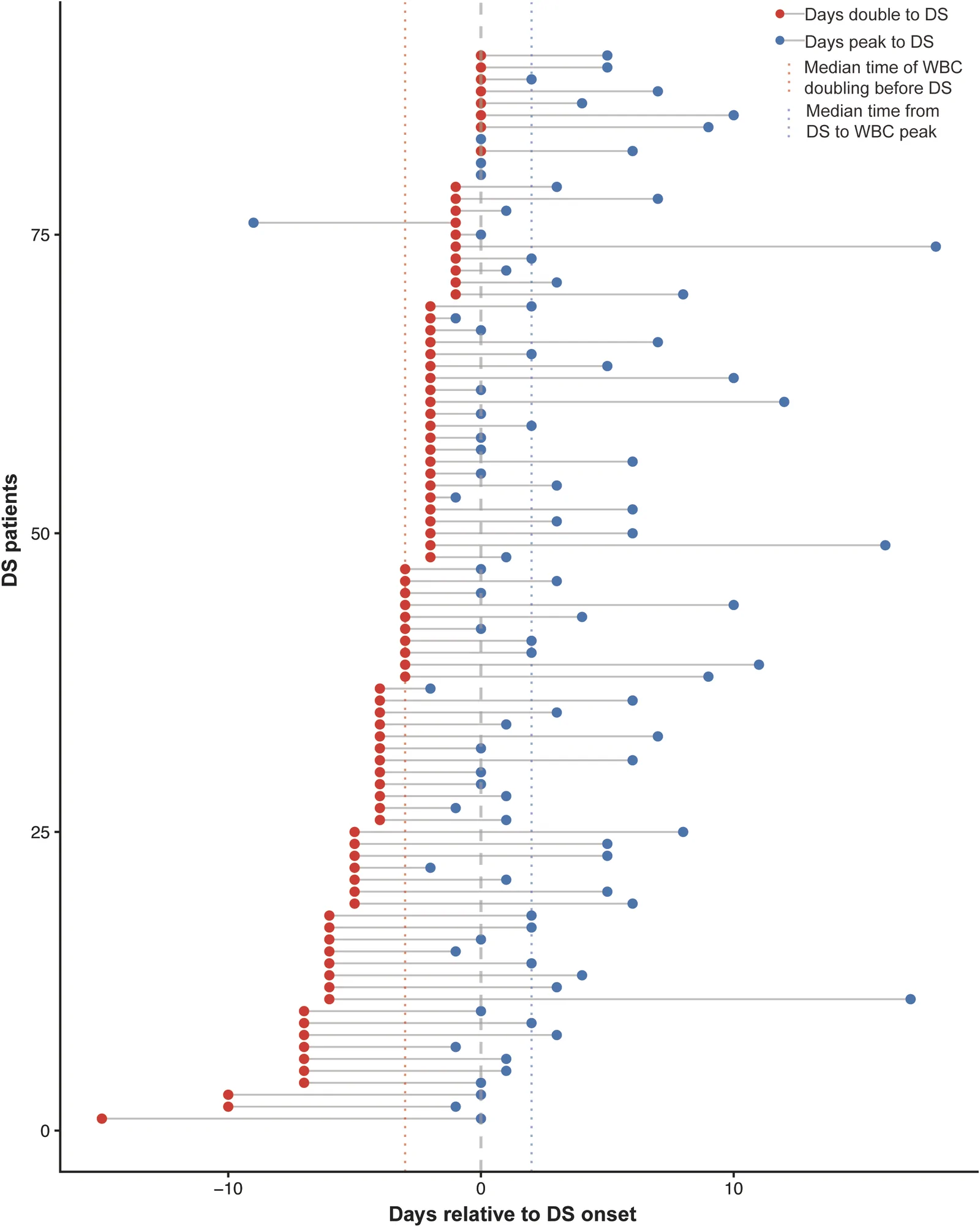
Timing of WBC doubling and WBC peak occurrence relative to DS onset.

### 3.5 Variable screening by LASSO regression

The remaining 12 clinical variables were enrolled in the LASSO regression analysis to reduce model overfitting and simplify the variable set. The optimal model performance was achieved at a tuning parameter λ of 0.0737. Ultimately, five variables with non-zero coefficients were identified as core predictive factors: rapid proliferation, WBC double group, PB promyelocyte percentage, WBC double days, and WBC doubling count. Rapid proliferation exhibited the largest positive coefficient, with a coefficient of 0.7788. WBC double days was the only variable with a negative predictive factor, with a coefficient of −0.0481. The remaining variables presented positive coefficients ranging from 0.0064 to 0.4226, indicating favorable predictive effects for DS occurrence (**Table 4**).

**Table 4:** Regression coefficients of variables selected by LASSO regression.

Variables	LASSO Coefficient (λ 1se = 0.0737)
Rapid proliferation	0.7788
WBC double group	0.4226
WBC Double Days	-0.0481
PB	0.0064
WBC Double	0.0202

PB, peripheral blood promyelocyte percentage, WBC, white blood cell.

### 3.6 Model construction using multivariable stepwise regression

The five key predictors with non-zero coefficients screened via LASSO dimensionality reduction were further incorporated into a multivariable stepwise logistic regression model to construct the final predictive model (**Table 5**). Rapid WBC proliferation (OR = 4.02, 95% CI: 1.90–8.89; P < 0.001) and elevated PB promyelocyte percentage (OR = 1.02, 95% CI: 1.00–1.03; P < 0.05) remained significant independent risk factors for DS. Extended WBC doubling days exerted a protective effect, with each one-day prolongation associated with a 19% reduction in DS risk. WBC doubling count was also included in the final stepwise model (OR = 1.06, 95% CI: 0.98–1.16; P > 0.05); however, it was not statistically significant and was therefore not retained as an independent predictive factor. All variables included in the final multivariable model had VIF values below 1.5 (range, 1.10–1.49), indicating no substantial multicollinearity.

**Table 5:** Multivariable logistic regression results.

Variables	OR	95% CI	P value
Rapid proliferation	4.02	1.90,8.89	<0.001
WBC Double Days	0.81	0.71,0.91	<0.001
PB	1.02	1.00,1.03	0.042
WBC _Double_	1.06	0.98,1.16	0.154

PB, peripheral blood; WBC, white blood cell.

### 3.7 Model validation

Internal validation of the established model was performed using ROC curve analysis, bootstrap calibration analysis, and DCA. The model demonstrated good discriminatory performance, with an AUC of 0.825 (95% CI: 0.770–0.880). The optimal probability threshold determined by the maximum Youden index was 0.38, corresponding to a sensitivity of 0.867 and a specificity of 0.701. After 1,000 bootstrap resampling procedures, the optimism-corrected AUC remained 0.817, indicating good model stability and discrimination.

The bootstrap calibration curve showed good agreement between the predicted and observed probabilities, with a mean absolute error of 0.074, suggesting satisfactory model calibration. DCA demonstrated that compared with the treat-none strategy, the prediction model provided greater net benefit across a range of threshold probabilities, particularly within the clinically relevant threshold range of 0.0–0.4, indicating its potential clinical utility.

Overall, the developed model showed good discrimination, calibration, and potential clinical utility. Based on the coefficients derived from the final multivariable logistic regression model, a nomogram was subsequently constructed to provide a visual tool for individualized DS risk prediction (**Figure 3**).

**Figure 3:**
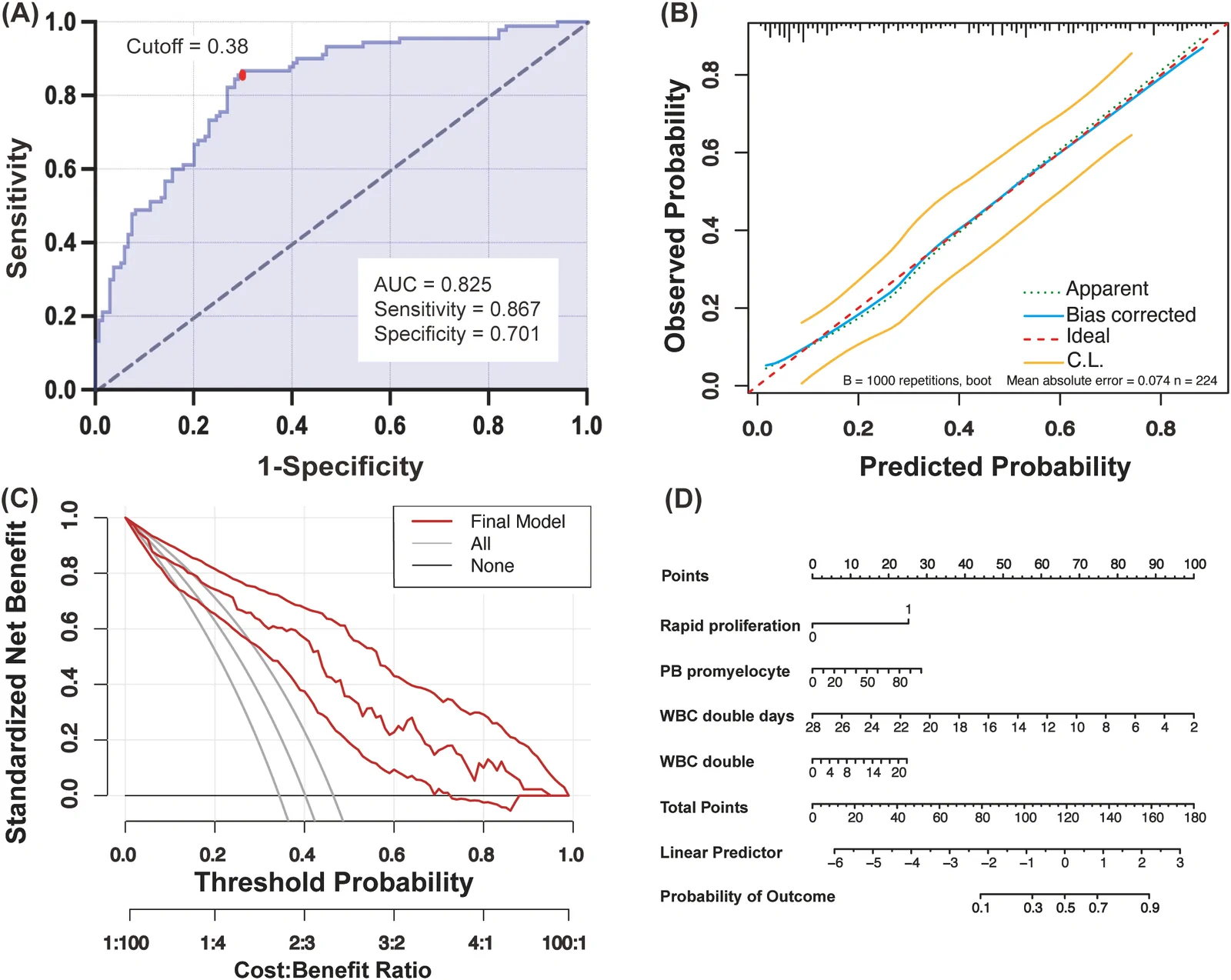
**(A)** ROC curve of the predictive model for DS. **(B)** Calibration curve of the prediction model for DS. **(C)** Decision curve analysis of the prediction model for DS. **(D)** Nomogram predicting DS in APL patients.

## 4 Discussion

We identified independent risk factors for DS and constructed an early prediction model using clinical data from 224 patients with low- and intermediate-risk APL. The DS incidence in our cohort (40.2%) was within the previously reported range of 2–50%; however, it was higher than that reported in many previous studies (1, 7–10). This discrepancy is likely attributable to differences in the study population and induction strategy. All included patients had low- or intermediate-risk APL and received single-agent ATO induction without routine prophylactic corticosteroids, which differed from the regimens used in most previous cohorts. Furthermore, current guidelines recommend prophylactic corticosteroids for patients with APL and elevated baseline WBC counts (24, 26). Nevertheless, prophylactic corticosteroids were not administered in the present cohort, given that all patients presented with baseline WBC counts < 10×10^9^/L. This variation in clinical management may further account for the relatively high DS incidence observed in our study.

Consistent with earlier reports (25, 27), compared with patients without DS, those with DS exhibited significantly higher baseline absolute counts of WBC, neutrophils and monocytes, and elevated BM and PB promyelocyte percentages. This finding indicates that a high baseline leukemic burden is a core risk factor for DS. ATO drives rapid promyelocyte differentiation, acutely increasing the circulating WBC burden. Meanwhile, APL cells secrete chemokines and upregulate cell adhesion molecules, which may trigger systemic inflammatory activation and multi-organ injury (11, 28, 29). APL cells that infiltrate the pulmonary microenvironment continuously release chemokines, contributing to uncontrolled pulmonary inflammation refractory to glucocorticoids, a key mechanism proposed to underlie steroid-resistant DS in clinical practice (12, 30). We also observed relatively severe coagulopathy in the DS group, including reduced fibrinogen, prolonged prothrombin time, elevated DIC scores, and an increased prevalence of overt DIC. These findings are consistent with published data (31) and suggest a close link between coagulation disorders and DS pathogenesis. APL cells disrupt physiological hemostasis and accelerate fibrinolysis, potentially exacerbating endothelial dysfunction and systemic inflammation and thereby contributing to DS onset. Coagulation parameters were excluded from the final model owing to collinearity. However, the utility of baseline coagulation profiles as adjunctive markers for DS risk stratification merits validation in prospective cohorts.

Several previous studies have identified BMI, BSA, and serum Cr as predictors of DS (17, 18, 32). However, no such associations were observed in our analysis. This inconsistency may be attributed to inter-cohort disparities in patient baseline characteristics or the limited sample size, although this interpretation requires confirmation in larger cohorts. Prior work has focused on the WBC peak as a predictive marker for DS, demonstrating elevated DS risk above threshold values of 31×10^9^/L or 43×10^9^/L (14–16). However, the WBC peak represents a post-treatment static endpoint that does not reflect early dynamic changes during induction therapy and cannot define the temporal relationship between WBC evolution and DS onset.

In our cohort, DS occurred predominantly within the first two weeks after ATO induction therapy, with a median onset time of 8 days (IQR, 7–11 days), consistent with previously reported onset times (10). Temporal analysis showed that WBC doubling preceded DS onset by a median of 3 days, whereas the WBC peak occurred after DS onset, with a median delay of 2.5 days. These findings suggest that the WBC peak represents a delayed marker with limited value for early DS risk assessment.

Based on these findings, we further investigated the association between early WBC kinetic patterns during ATO induction and DS development. Rapid WBC proliferation, PB promyelocyte percentage, and WBC doubling time were associated with DS occurrence. Among these variables, rapid WBC proliferation and an elevated PB promyelocyte proportion were identified as independent risk factors, whereas prolonged WBC doubling time was associated with a reduced risk of DS. Patients with WBC doubling within 6.5 days or an elevated proliferation rate had an increased risk of DS, consistent with previous domestic and international studies (7, 21, 33). These findings indicate that early WBC kinetic characteristics can provide additional information beyond conventional WBC parameters and serve as relatively time-sensitive indicators for early DS risk assessment.

Based on these characteristics, we developed a nomogram for early DS risk stratification in patients with low- and intermediate-risk APL. The model incorporated rapid WBC proliferation, PB promyelocyte percentage, and WBC doubling time; it showed good predictive performance, with an AUC of 0.825. After bootstrap internal validation, the optimism-corrected AUC remained 0.817. Calibration analysis demonstrated good agreement between the predicted and observed DS probabilities, whereas DCA indicated the model’s potential clinical net benefit across relevant threshold probabilities. At the selected cutoff, the model achieved a sensitivity of 0.867 and a specificity of 0.701, indicating that both false-positive and false-negative classifications remain possible. False-positive classifications may result in unnecessary intensified monitoring, whereas false-negative classifications may delay the recognition of patients at risk for DS. Therefore, the model should be used as an adjunctive tool for early risk stratification and interpreted together with clinical assessment and routine monitoring, rather than as a stand-alone diagnostic tool or the sole basis for therapeutic decisions.

Unlike risk assessment approaches mainly based on baseline clinical characteristics or static laboratory measurements, our model was developed specifically for patients with low- and intermediate-risk APL receiving single-agent ATO induction; it also incorporated serial WBC kinetic parameters during the early induction phase. This approach characterizes the temporal relationship between WBC changes and DS development and provides a quantitative tool for early DS risk assessment and dynamic patient monitoring.

This study has some limitations. First, as a single-center retrospective study, it is susceptible to potential selection and information biases. Although internal validation using bootstrap resampling demonstrated robust model performance, external validation in independent multicenter cohorts was not performed in the present study. In addition, exclusion of patients who died before completing the predefined 14-day observation period may have introduced selection bias by underrepresenting patients with severe early clinical courses. Future prospective studies involving larger and geographically diverse populations are warranted to externally validate and further refine the model. Second, our model was developed exclusively in patients with low- and intermediate-risk APL receiving single-agent ATO induction therapy, without concomitant ATRA. With the increasing use of ATRA-containing regimens, particularly ATRA plus ATO, differences in induction therapy may influence early WBC kinetics and the pattern of DS occurrence. Previous studies have also suggested that early leukocytosis during ATRA plus ATO treatment may exhibit distinct differentiation patterns and is associated with DS development (7). Therefore, the predictive performance and thresholds derived from the present cohort should not be directly extrapolated to patients receiving ATRA plus ATO. Future studies should further evaluate the association between these WBC kinetic characteristics and DS risk in independent cohorts receiving ATRA plus ATO, externally validate the present model, and, if necessary, recalibrate it according to the treatment setting. Third, some clinical manifestations of DS are nonspecific, particularly fever, which may overlap with infectious conditions and potentially lead to diagnostic misclassification or overdiagnosis. Although infection was clinically evaluated based on the patients’ manifestations and available laboratory and microbiological findings, and isolated single manifestations were not classified as DS, the retrospective nature of this study precluded the complete exclusion of diagnostic misclassification arising from overlapping features between DS and infection.

In conclusion, early WBC kinetic characteristics, including rapid WBC proliferation and WBC doubling time, were associated with DS development in patients with low- and intermediate-risk APL. Compared with the WBC peak, these dynamic parameters provided earlier information for DS risk assessment. The nomogram, which incorporates early WBC kinetics and clinical characteristics, showed good predictive performance and may serve as a quantitative tool for early DS risk stratification and clinical monitoring.

## Data Availability

The raw data supporting the conclusions of this article will be made available by the authors, without undue reservation.
